# Supplementary material for: Microglia contribute to neuronal synchrony despite endogenous ATP-related phenotypic transformation in acute mouse brain slices
Source: Nat Commun. 2024 Jun 26;15:5402. doi: 10.1038/s41467-024-49773-1 (PMC11208608; doi:10.1038/s41467-024-49773-1)
Supplement: Supplementary file 1 — Supplementary Information [file 41467_2024_49773_MOESM1_ESM.pdf]

Supplementary Figures

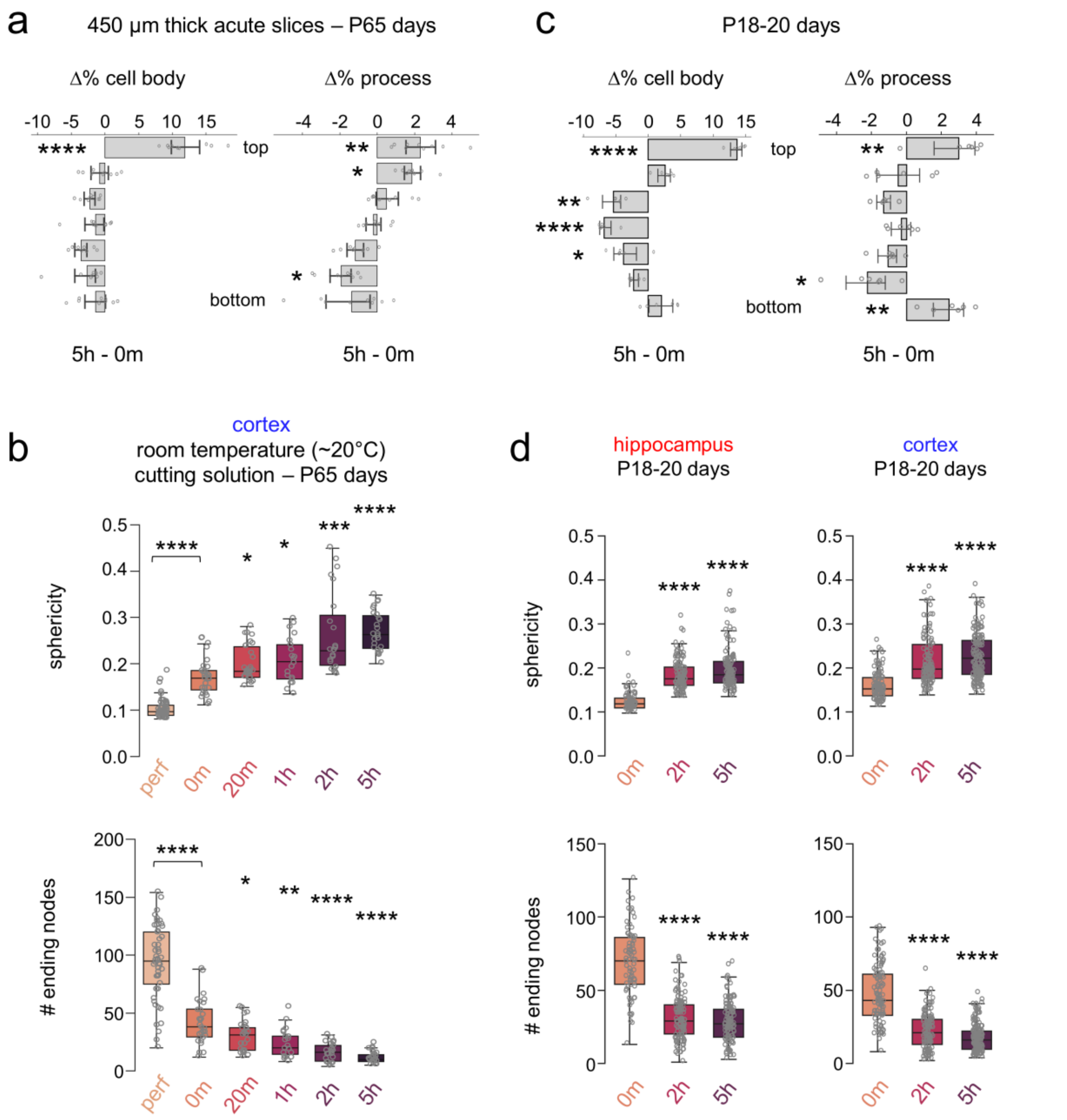

Supplementary Figure 1. Microglial migration is also prevalent in thick slices, and characteristic morphological changes are not mitigated by room temperature cutting solution

a. Bar plots representing measured changes of microglial cell body numbers (left) and area covered by processes (right) in percentages calculated between 0 min and 5 hours across the top and bottom layers of acute slice preparations (~450 μm thick). N=3 animals, n=3 slice/animal, P65 days, mean ± SEM, two-way ANOVA with Tukey's multiple comparison test, $p<0.0001$.

b. Quantification of extracted morphological features from acute slices prepared with room temperature standard ACSF cutting solution, regarding sphericity in cortex (left) and in hippocampus (right). N=3 animals, P65 days, one-way ANOVA with Dunnett's multiple comparison test; asterisks indicate comparison with perfused 0 min values, $p<0.0001$.

c. Bar plots representing measured changes of microglial cell body numbers (left) and area covered by processes (right) in percentages calculated between 0 min and 5 hours across the top and bottom layers of acute slice preparations (~450 µm thick). N=3 animals, n=3 slice/animal, P19 days, mean ± SEM, two-way ANOVA with Tukey's multiple comparison test, $p<0.0001$.
d. Quantification of extracted morphological features from acute slices regarding sphericity in cortex (left) and in hippocampus (right). N=3 animals, P19 days, one-way ANOVA with Dunnett's multiple comparison test; asterisks indicate comparison with perfused 0 min values, $p<0.0001$.

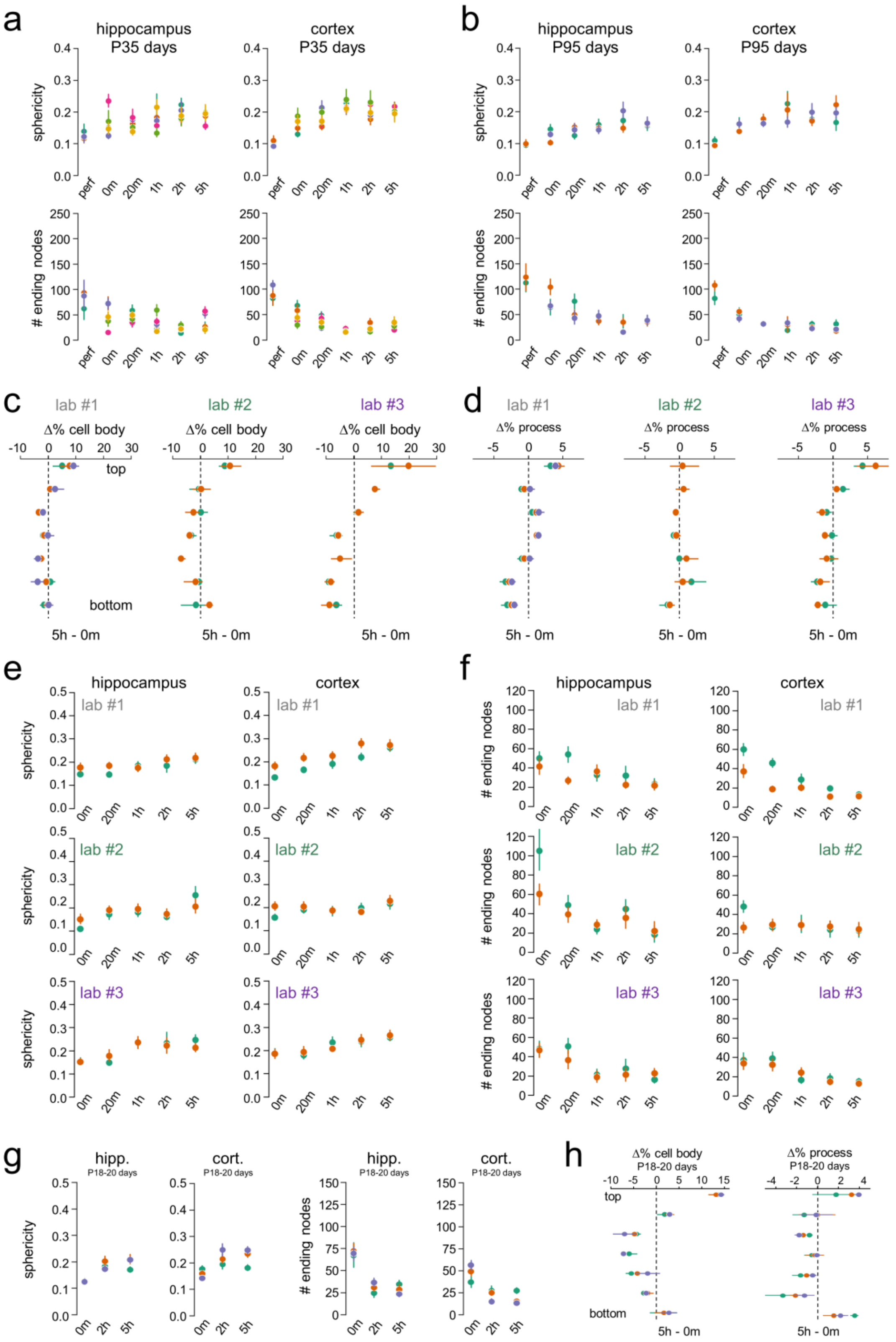

Supplementary Figure 2. Microglial migration and morphological changes in in individual animals

a. Upper panels: quantification of extracted morphological features regarding sphericity in cortex and in hippocampus. Lower panels: quantification of extracted morphological features regarding

number of process endings / cell in the hippocampus and cortex. N=8 animal (3 perfused, 5 immersion fixed), P35 days. Different colours represent different animals.

b. Upper panels: quantification of extracted morphological features regarding sphericity in cortex and in hippocampus. Lower panels: quantification of extracted morphological features regarding number of process endings / cell in the hippocampus and cortex. N=5 animal (2 perfused, 5 immersion fixed), P95 days. Different colours represent averages for different animals.
c. Measured changes of microglial cell body numbers in percentages calculated between 0 min and 5 hours across the top and bottom layers of acute slice preparations. N=3 animal/lab1; 2 animal/lab#2; 2 animal/lab#3; n=3 slices/animal, P65 days. Different colours represent averages for different animals.
d. Measured changes of microglial process distributions in percentages calculated between 0 min and 5 hours across the top and bottom layers of acute slice preparations. N=3 animal/lab1; 2 animal/lab#2; 2 animal/lab#3; n=3 slices/animal, P65 days. Different colours represent averages for different animals.
e. Quantification of extracted morphological features across different laboratories regarding sphericity in hippocampus (left) and in cortex (right). N=3 animal/lab1; 2 animal/lab#2; 2 animal/lab#3; n=3 slices/animal, P65 days. Different colours represent averages for different animals.
f. Same as in d, regarding number of ending nodes/cell in cortex (left) and in hippocampus (right). N=3 animal/lab1; 2 animal/lab#2; 2 animal/lab#3; n=3 slices/animal, P65 days. Different colours represent averages for different animals.
g. Quantification of extracted morphological features from acute slices regarding sphericity in cortex (left) and in hippocampus (right). N=3 animals, P19 days. Different colours represent averages for different animals.
h. Bar plots representing measured changes of microglial cell body numbers (left) and area covered by processes (right) in percentages calculated between 0 min and 5 hours across the top and bottom layers of acute slice preparations (~450 µm thick). N=3 animals, n=3 slice/animal, P19 days. Different colours represent averages for different animals.

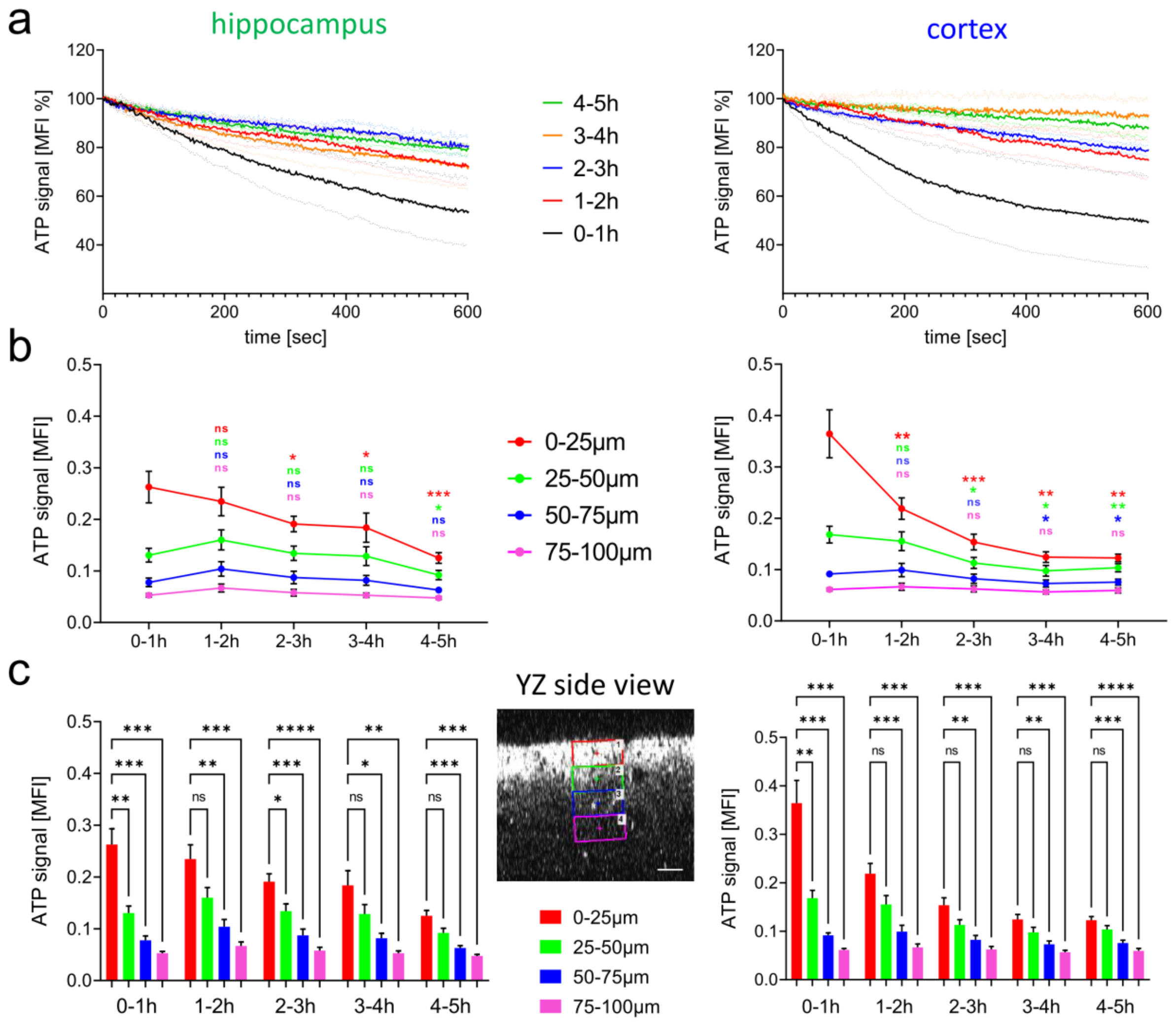

Supplementary Figure 3. Characteristics of extracellular ATP signal changes in acute brain slices

a. MFI data collected from hippocampal or cortical slices imaged 1-2h (red), 2-3h (blue), 3-4h (orange) or 4-5 (green) hours after slice preparation show significantly slower ATP reduction, than those from the earliest timepoints (0-1h, black). Note, that data were collected from regions of interests where no apparent flashing ATP activity was observed. Two-way ANOVA, $p<0.0001$ for all (red, blue, orange, green) compared to 0-1h (black).
b. MFI data obtained from Z stacks. ATP signal intensity and subsequent reduction over time is most apparent next to the slice surface (0-25, 25-50µm) compared to deeper layers (75-100µm). N=3 animals, n=5 slices/animal. Two-way repeated measures ANOVA, Dunnett's multiple comparison test, cortex $F(12,128)=19.48$, $p<0,001$, HC $F(12,128)=8,706$, $p<0,001$.
c. Gradient in ATP signal intensity from the slice surface to the ~100 µm depth of the slice is maintained for at least 5 hours after slice preparation. N=3 animals, n=5 slices/animal. Two-way repeated measures ANOVA, Dunnett's multiple comparison test, ROI size: 25 *50 µm, Scale bar: 25 µm.

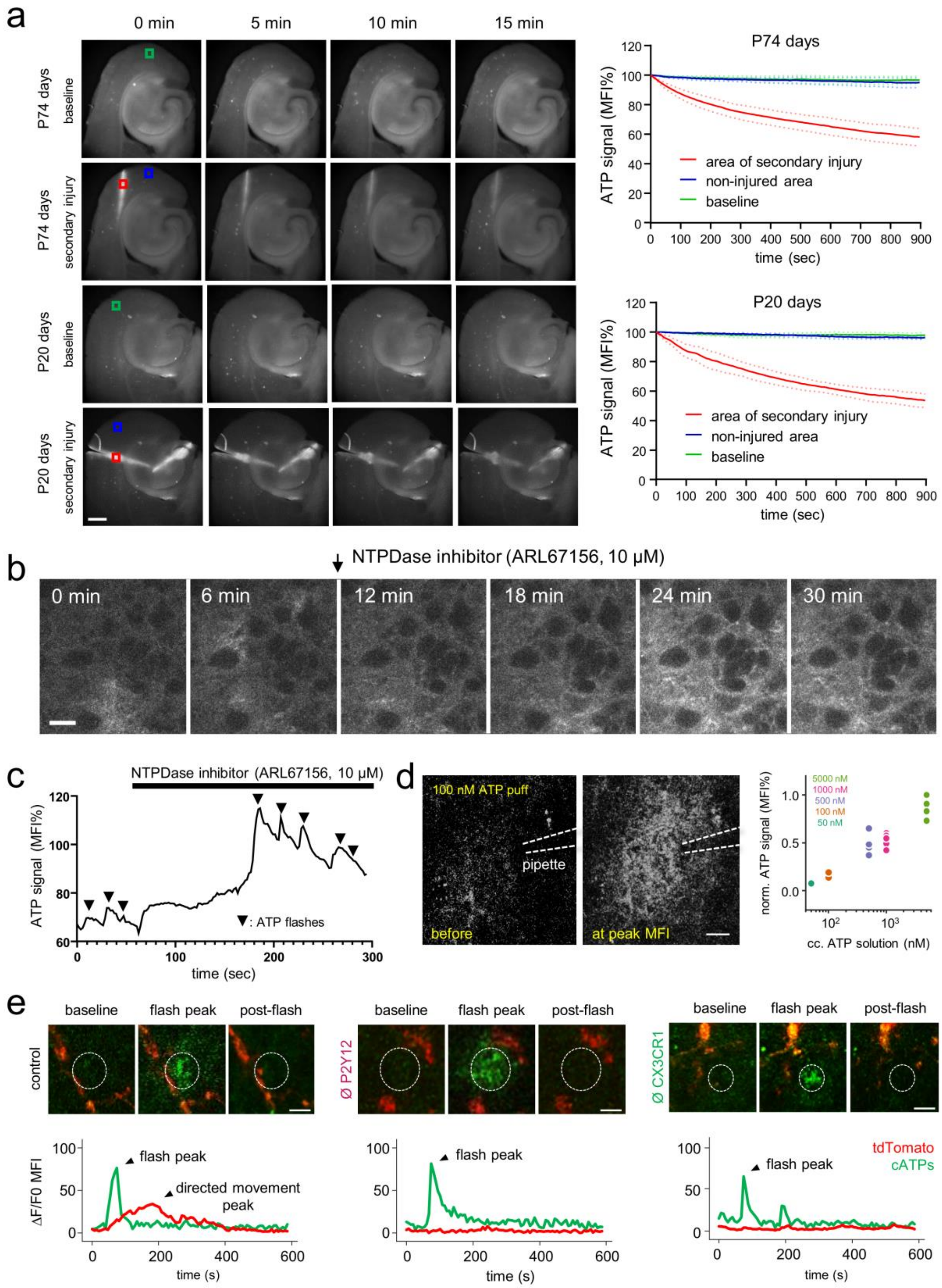

Supplementary Figure 4. Assessment of ATP sensor signal and calibrating intensity

a. Secondary mechanical injuries result in immediate, intense ATP release (red) compared to non-injured sites (blue) or non-injured slices (green) recorded ~4 hours after slice preparation. Note the significant difference in baseline fluorescent intensity and signal decay (right), which develops similarly in slices from both P74 or younger, P20 mice. N=5 animals, n=3 slices/animal.

Two-way repeated measures ANOVA with Geisser-Greenhouse correction, **$p<0.01$ for secondary injury vs non injured areas and not significant for non-injured areas in injured vs non injured slices. Scale bar: 500 μm.

b. Representative images on the effect of NTPDase inhibitor (ARL67156, 10 μM), resulting in gradual increase of ATP signal. Note, that NTPDase blockade does not abolish flashing ATP release activity. Scale bar: 20 μm.
c. Graph showing the effect of NTPDase inhibitor (ARL67156, 10 μM), resulting in gradual increase of ATP signal. Note, that NTPDase blockade does not abolish flashing ATP release activity.
d. ATP sensor signal before and after a puff of 100 nM ATP diluted in standard ACSF (left). The measured normalized MFI signal of puffs elicited with different ATP concentrations indicates that the ATP sensor scales linearly with ATP concentration. Scale bar: 5 μm.
e. Representative images (top) of flash ATP events (green) and microglia process (tdTomato, red) displacement under control conditions or after P2Y12R or CX3CR1 blockade. Images were taken from Suppl. Video 7. ROIs indicate ATP event areas at maximum intensity. Representative ΔF/F traces of ATP sensor activity (green) and superimposed microglia process accumulation (tdTomato, red) within the ATP flash territories. Scale bar: 5 μm.

a

	inflare incidence [number of events/10min]	peak intensity [MFI]	peak area [µm^2]	peak duration [sec]	rise time [sec]	decay time [sec]
hippocampus	12.3 ± 2.2 n=15	116.1 ± 10.2 n=181	229.1 ± 16.5 n=151	88.9 ± 5.9 n=156	15.02 ± 1.4 n=178	75.8 ± 5.3 n=155
cortex	25.4 ± 5.2 n=15	200.8 ± 11.6 n=360	243.1 ± 13.9 n=309	81.9 ± 3.8 n=318	12.2 ± 0.7 n=369	70.6 ± 3.4 n=318
	unpaired t-test *p=0.0258	Mann-Whitney test ****p<0.0001	Mann-Whitney test p=0.3912	Mann-Whitney test p=0.3803	Mann-Whitney test p=0.2474	Mann-Whitney test p=0.4207

b

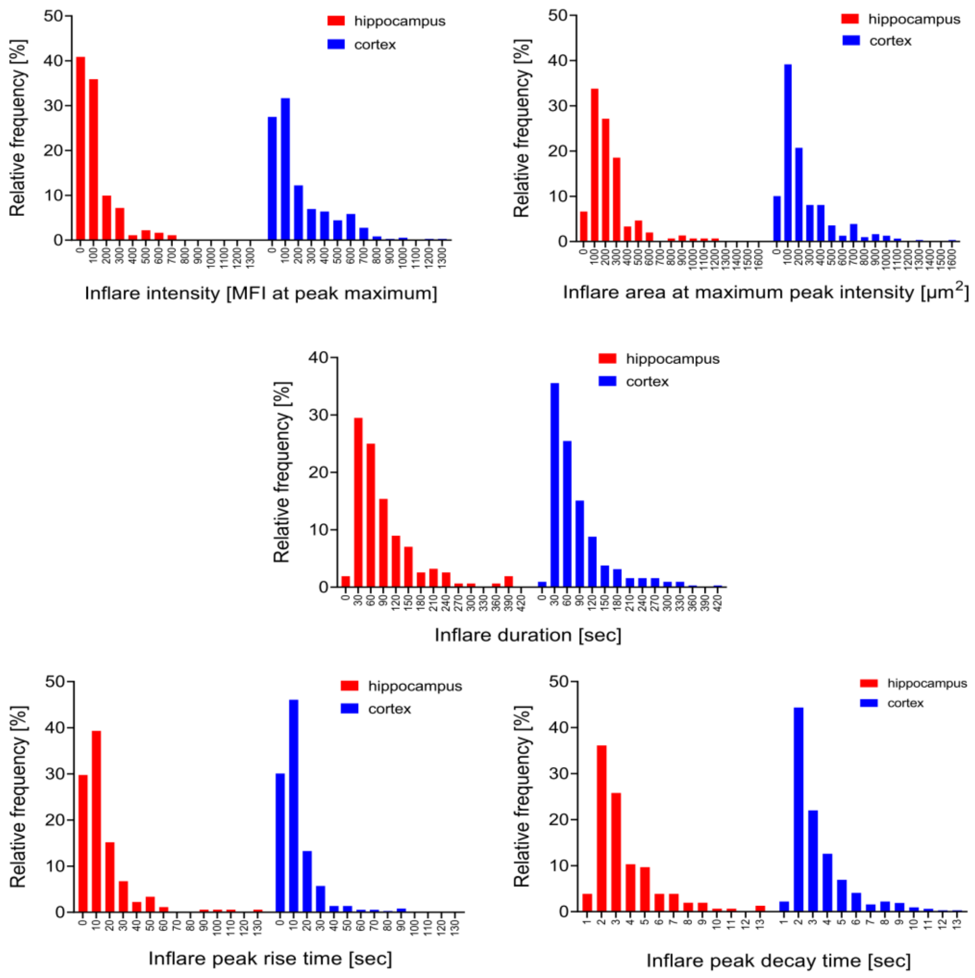

Supplementary Figure 5. Properties of ATP flash events in acute slices

a. Statistics of ATP flash properties.
b. Histograms show data distribution of flash intensity, area, duration, rise time and decay time.

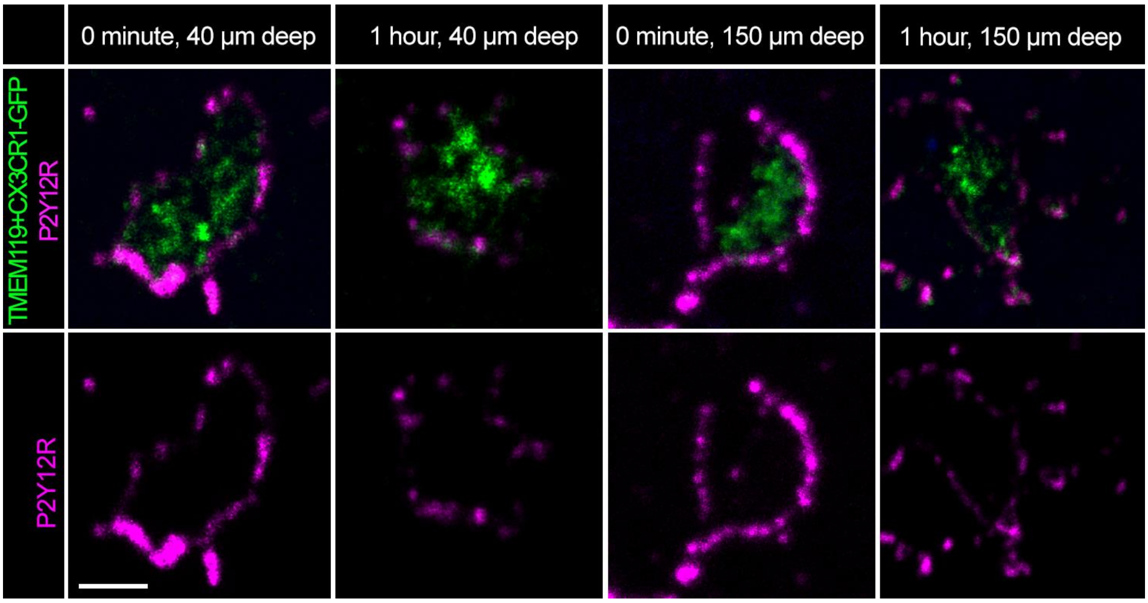

Supplementary Figure 6. Rapid downregulation of P2Y12R on microglia during the incubation process at different depths from the slice surface

Representative images of microglial cell bodies from 40 µm and 150 µm depths from slice surface at 0 minutes and after 1 hours incubation. TMEM119 and CX3CR1-GFP (both in green) labelling is shown together with the magenta colored P2Y12R labelling (top row) and P2Y12R labelling only (bottom row). The quantitative post-embedding technique shows that incubation time dependent P2Y12R downregulation is not affected by depth within the slices. Scale bar: 3 µm.

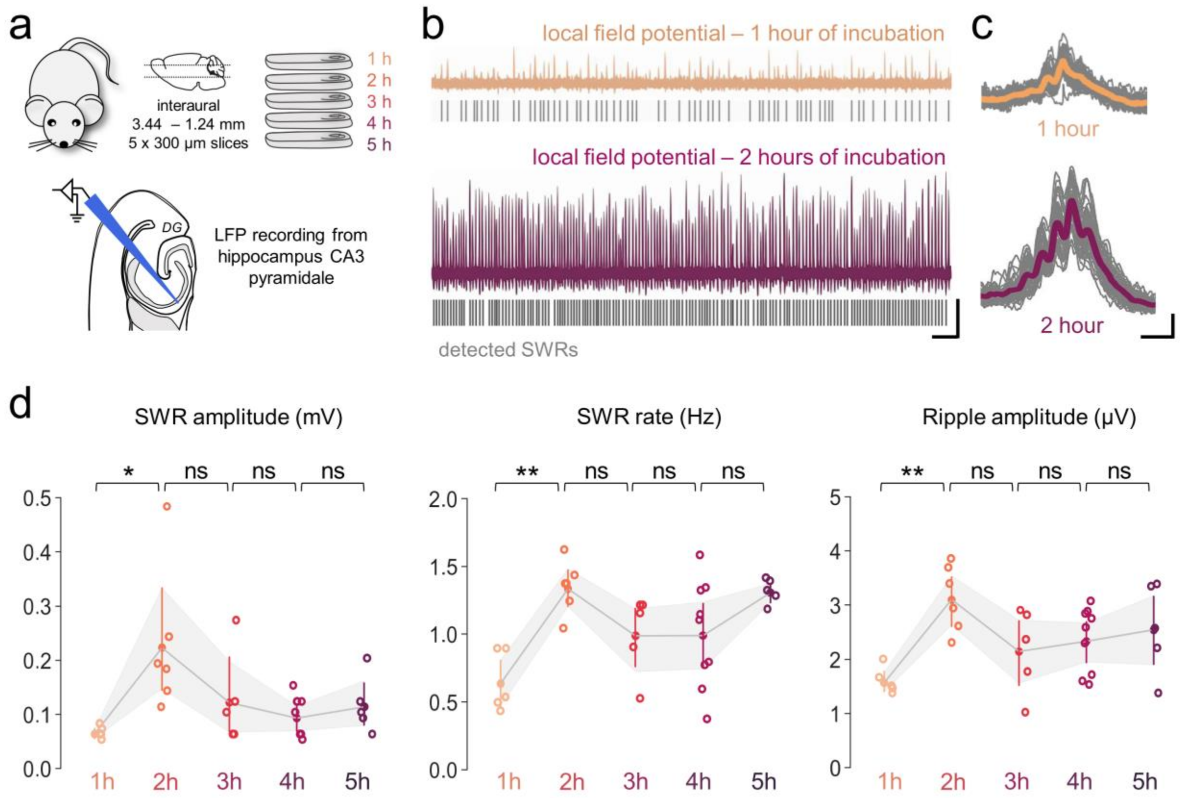

Supplementary Figure 7. Temporal course of the emergence of sharp wave-ripple activity after acute slice preparation

a. Schematic representation of the experiment. CX3CR1$^{+/GFP}$ littermates (N=8; P65 days) were used to create acute hippocampal slice preparations and placed into an interface-type incubation chamber for at least 1 hour of recovery time. Subsequently, slices were transferred at specific timepoints (hourly after 1-5 hours) into a recording chamber to measure sharp-wave ripple (SWR) activity via local field potential recordings (LFP) registered from the CA3 pyramidal layer of the hippocampus.
b. Representative LFP recordings measured after 1 (yellow) or 2 hours (purple) of incubation. Grey lines represent detected SWR events (bars 10 s, 50 µV).
c. Representative averaged traces of detected SWRs (N=#50 in total) measured after 1 hour (yellow: average, grey: individual events) or 2 hours (purple: average, grey: individual events) of incubation (bars 100 ms, 25 µV).
d. Quantification of SWR amplitude (left), rate (right) and Ripple amplitude (right) comparing events measured after different time spent in the incubation chamber before recording. N=8 animals, P65 days; One-way ANOVA with Tukey’s multiple comparison test, $p<0.05$ (left), $p<0.01$ (middle), $p<0.05$ (right).

Supplementary Tables

Supplementary Table 1: List of antibodies used in the study.

Primary antibodies	host	source	catalog nr.	dilution	RRID
Gephyrin	Mouse	Synaptic Systems	147 021	1:500	AB_2232546
GFP	Chicken	Invitrogen	A10262	1:1000	AB_2534023
Homer1	Rabbit	Synaptic Systems	160 003	1:500	AB_887730
IBA1	Guinea pig	Synaptic Systems	234004	1:500	AB_2493179
Kv2.1	Mouse	NeuroMab	75-014	1:500	AB_10673392
P2Y12R	Rabbit	Anaspec	AS-55043A	1:2000	AB_2298886
TMEM119	Chicken	Synaptic Systems	400 006	1:500	AB_2744643
vGAT	Guinea pig	Synaptic Systems	131 004	1:500	AB_887873
VGLUT1	Guinea pig	Synaptic Systems	135 304	1:1000	AB_887878
**Secondary antibodies**	**host**	**source**	**catalog nr.**	**dilution**	**RRID**
Alexa 488 Streptavidin	-	-	S-11223	1:400	-
Alexa 488 anti-chicken	donkey	Jackson ImmunoResearch Labs	703-546-155	1:400	AB_2340376
Alexa 488 anti-guinea-pig	donkey	Jackson ImmunoResearch Labs	706-546-148	1:500	AB_2340473
Alexa 488 anti-guinea-pig	donkey	Jackson ImmunoResearch Labs	706-546-148	1:500	AB_2340473
Alexa 488 anti-mouse	donkey	Thermo Fisher Scientific	A-21202	1:500	AB_141607
Alexa 488 anti-rabbit	donkey	Jackson	711-546-152	1:500	AB_2340619
Alexa 594 anti-guinea-pig	goat	LifeTech	A11076	1:500	AB_141930
Alexa 594 anti-mouse	donkey	Invitrogen	A-21203	1:500	AB_141633
Alexa 594 anti-rabbit	donkey	LifeTech	A21207	1:500	AB_141637
Alexa 647 anti-chicken	donkey	Jackson ImmunoResearch Labs	703-606-155	1:500	AB_2340380
Alexa 647 anti-rabbit	donkey	Jackson ImmunoResearch Labs	711-605-152	1:400-500	AB_2492288
Alexa 647anti-guinnea-pig	donkey	Jackson ImmunoResearch Labs	706-606-148	1:500	AB_2340477
Biotinylated anti-chicken	goat	Vector Laboratories	BA-9010	1:400	AB_2336114

Supplementary Table 2: Statistical Test Results, Figure 2

		hippocampus, P35	cortex, P35
sphericity	F(Dfn, DFd)	19.88(5, 521.5)	59.81(5, 716.5)
	p value	<0.0001	<0.0001
# ending nodes	F(Dfn, DFd)	30.04(5, 117.2)	116.7(5, 205.6)
	p value	<0.0001	<0.0001
		**hippocampus, P95**	**cortex, P95**
sphericity	F(Dfn, DFd)	26.20(5, 162.4)	41.44(5, 165.9)
	p value	<0.0001	<0.0001
# ending nodes	F(Dfn, DFd)	40.71(5, 96.20)	103.8(5, 157.1)
	p value	<0.0001	<0.0001
		**ctrl vs. P2Y12 KO**	**ctrl vs. CX3CR1 KO**
sphericity	F(Dfn, DFd)	10.57(2, 259)	3.175(2, 309)
	p value	<0.0001	0.043
# ending nodes	F(Dfn, DFd)	18.26(2, 401)	0.7121(2, 309)
	p value	<0.0001	0.4914

Supplementary Table 3: Statistical Test Results, Figure 3

hippocampus		lab #1	lab #2	lab #3
**sphericity**	**F(Dfn, DFd)**	11.51(4, 179.9)	15.23(4, 151.1)	22.48(4, 132.4)
	**p value**	<0.0001	<0.0001	<0.0001
**# ending nodes**	**F(Dfn, DFd)**	14.48(4, 203.8)	30.81(4, 105.5)	29.50(4, 170.7)
	**p value**	<0.0001	<0.0001	<0.0001
**cortex**		**lab #1**	**lab #2**	**lab #3**
**sphericity**	**F(Dfn, DFd)**	45.73(4, 309.9)	6.431(4, 238)	20.99(4, 268.2)
	**p value**	<0.0001	<0.0001	<0.0001
**# ending nodes**	**F(Dfn, DFd)**	65.98(4, 201.7)	6.613(4, 246.9)	32.61(4, 203.2)
	**p value**	<0.0001	<0.0001	<0.0001
		**lab #1**	**lab #2**	**lab #3**
**Δ% cell body**	**F(Dfn, DFd)**	2.305(24, 408)	3.668(24, 150)	4.440(24, 150)
	**p value**	0.0005	<0.0001	<0.0001
**Δ% process**	**F(Dfn, DFd)**	4.766(24, 408)	2.756(24, 150)	2.008(24, 150)
	**p value**	<0.0001	<0.0001	0.0063
