# Supplementary material for: Microglia contribute to neuronal synchrony despite endogenous ATP-related phenotypic transformation in acute mouse brain slices
Source: Nat Commun. 2024 Jun 26;15:5402. doi: 10.1038/s41467-024-49773-1 (PMC11208608; doi:10.1038/s41467-024-49773-1)
Supplement: Supplementary file 3 — Reporting Summary [file 41467_2024_49773_MOESM3_ESM.pdf]

nature portfolio

Corresponding author(s): Ádám Dénes

Last updated by author(s): May 17.24.

Reporting Summary

Nature Portfolio wishes to improve the reproducibility of the work that we publish. This form provides structure for consistency and transparency in reporting. For further information on Nature Portfolio policies, see our Editorial Policies and the Editorial Policy Checklist.

Statistics

For all statistical analyses, confirm that the following items are present in the figure legend, table legend, main text, or Methods section.

n/a	Confirmed	
☐	☒	The exact sample size (*n*) for each experimental group/condition, given as a discrete number and unit of measurement
☐	☒	A statement on whether measurements were taken from distinct samples or whether the same sample was measured repeatedly
☐	☒	The statistical test(s) used AND whether they are one- or two-sided<br>*Only common tests should be described solely by name; describe more complex techniques in the Methods section.*
☐	☒	A description of all covariates tested
☐	☒	A description of any assumptions or corrections, such as tests of normality and adjustment for multiple comparisons
☐	☒	A full description of the statistical parameters including central tendency (e.g. means) or other basic estimates (e.g. regression coefficient) AND variation (e.g. standard deviation) or associated estimates of uncertainty (e.g. confidence intervals)
☐	☒	For null hypothesis testing, the test statistic (e.g. *F*, *t*, *r*) with confidence intervals, effect sizes, degrees of freedom and *P* value noted<br>*Give P values as exact values whenever suitable.*
☒	☐	For Bayesian analysis, information on the choice of priors and Markov chain Monte Carlo settings
☒	☐	For hierarchical and complex designs, identification of the appropriate level for tests and full reporting of outcomes
☒	☐	Estimates of effect sizes (e.g. Cohen's *d*, Pearson's *r*), indicating how they were calculated

Our web collection on statistics for biologists contains articles on many of the points above.

Software and code

Policy information about availability of computer code

Data collection	NIS-Elements AR Analysis Version 5.00.00<br>ImageJ Version 1.52r(NIH, US)<br>MATLAB-based Microglia Morphology Quantification:<br>https://github.com/isdneuroimaging/mmqt<br>Electrophysiology recording software:<br>custom software developed in C#.NET and VB.NET
Data analysis	MS Office 2016<br>Matlab Version R2016a (Mathwork, US)<br>FIJI (Image J Version 1.5 3f, NIH, US)<br>NIS-Elements AR Analyzis Version 5.00.00<br>Electrophysiology and Statistical analysis:<br>custom program developed in Python environment 2.7.0<br>Prism 10 for Windows Version 10.1.1 (323)

For manuscripts utilizing custom algorithms or software that are central to the research but not yet described in published literature, software must be made available to editors and reviewers. We strongly encourage code deposition in a community repository (e.g. GitHub). See the Nature Portfolio guidelines for submitting code & software for further information.

Data

Policy information about availability of data

All manuscripts must include a data availability statement. This statement should provide the following information, where applicable:

- Accession codes, unique identifiers, or web links for publicly available datasets
- A description of any restrictions on data availability
- For clinical datasets or third party data, please ensure that the statement adheres to our policy

The datasets generated and analysed during the current paper are available from the corresponding author on reasonable request.

Research involving human participants, their data, or biological material

Policy information about studies with human participants or human data. See also policy information about sex, gender (identity/presentation), and sexual orientation and race, ethnicity and racism.

Reporting on sex and gender	*Use the terms sex (biological attribute) and gender (shaped by social and cultural circumstances) carefully in order to avoid confusing both terms. Indicate if findings apply to only one sex or gender; describe whether sex and gender were considered in study design; whether sex and/or gender was determined based on self-reporting or assigned and methods used. Provide in the source data disaggregated sex and gender data, where this information has been collected, and if consent has been obtained for sharing of individual-level data; provide overall numbers in this Reporting Summary. Please state if this information has not been collected. Report sex- and gender-based analyses where performed, justify reasons for lack of sex- and gender-based analysis.*
Reporting on race, ethnicity, or other socially relevant groupings	*Please specify the socially constructed or socially relevant categorization variable(s) used in your manuscript and explain why they were used. Please note that such variables should not be used as proxies for other socially constructed/relevant variables (for example, race or ethnicity should not be used as a proxy for socioeconomic status). Provide clear definitions of the relevant terms used, how they were provided (by the participants/respondents, the researchers, or third parties), and the method(s) used to classify people into the different categories (e.g. self-report, census or administrative data, social media data, etc.) Please provide details about how you controlled for confounding variables in your analyses.*
Population characteristics	*Describe the covariate-relevant population characteristics of the human research participants (e.g. age, genotypic information, past and current diagnosis and treatment categories). If you filled out the behavioural & social sciences study design questions and have nothing to add here, write "See above."*
Recruitment	*Describe how participants were recruited. Outline any potential self-selection bias or other biases that may be present and how these are likely to impact results.*
Ethics oversight	*Identify the organization(s) that approved the study protocol.*

Note that full information on the approval of the study protocol must also be provided in the manuscript.

Field-specific reporting

Please select the one below that is the best fit for your research. If you are not sure, read the appropriate sections before making your selection.

☒ Life sciences ☐ Behavioural & social sciences ☐ Ecological, evolutionary & environmental sciences

For a reference copy of the document with all sections, see nature.com/documents/nr-reporting-summary-flat.pdf

Life sciences study design

All studies must disclose on these points even when the disclosure is negative.

Sample size	Sample size was determined by a priori power calculation using G*Power 3.1.9.2 software with mean differences and 20-25% standard deviations based on pilot studies (power 80%, α 0.05).
Data exclusions	In the cell translocation measurement slices of one animal were insufficiently fixed due to technical issues, and were excluded from the analysis pre hoc.
Replication	To ensure the best reproducibility of anatomical data, all analysis have been performed in a blinded manner, from multiple sets of experiments. Electrophysiological measurements were performed in multiple sets of experiments, with experimental sessions separated by several weeks.
Randomization	Animals were randomly selected for experimental groups, where applicable.
Blinding	Analysis was performed in a strictly blinded manner to ensure unbiased results. Datasets were analysed by multiple observers, who did not know the experimental group the dataset belonged to. Datasets and image files were given random codes before the analysis process by

someone not involved in the analysis itself.

Reporting for specific materials, systems and methods

We require information from authors about some types of materials, experimental systems and methods used in many studies. Here, indicate whether each material, system or method listed is relevant to your study. If you are not sure if a list item applies to your research, read the appropriate section before selecting a response.

Materials & experimental systems

n/a	Involved in the study	
☐	☒	Antibodies
☒	☐	Eukaryotic cell lines
☒	☐	Palaeontology and archaeology
☐	☒	Animals and other organisms
☒	☐	Clinical data
☒	☐	Dual use research of concern
☒	☐	Plants

Methods

n/a	Involved in the study	
☒	☐	ChIP-seq
☒	☐	Flow cytometry
☒	☐	MRI-based neuroimaging

Antibodies

Antibodies used

Gephyrin Mouse Synaptic Systems 147 021 AB_2232546
GFP Chicken lnvitrogen A10262 AB_2534023
Homerl Rabbit Synaptic Systems 160 003 AB_887730
IBAl Guinea pig Synaptic Systems 234004 AB_2493179
Kv2.1 Mouse NeuroMab 75-014 AB_10673392
P2Y12R Rabbit Anaspec AS-55043A AB_2298886
TMEM119 Chicken Synaptic Systems 400 006 AB_2744643
vGAT Guinea pig Synaptic Systems 131004 AB_887873
VGLUTl Guinea pig Synaptic Systems 135 304 AB_887878
Alexa 488 Streptavidin - - S-11223 -
Alexa 488 anti-chicken donkey Jackson lmmunoResearch Labs 703-546-155 AB_2340376
Alexa 488 anti-guinea-pig donkey Jackson lmmunoResearch Labs 706-546-148 AB_2340473
Alexa 488 anti-guinea-pig donkey Jackson lmmunoResearch Labs 706-546-148 AB_2340473
Alexa 488 anti-mouse donkey Thermo Fisher Scientific A-21202 AB_141607
Alexa 488 anti-rabbit donkey Jackson 711-546-152 AB_2340619
Alexa 594 anti-guinea-pig goat LifeTech A11076 AB_141930
Alexa 594 anti-mouse donkey lnvitrogen A-21203 AB_141633
Alexa 594 anti-rabbit donkey LifeTech A21207 AB_141637
Alexa 647 anti-chicken donkey Jackson lmmunoResearch Labs 703-606-155 AB_2340380
Alexa 647 anti-rabbit donkey Jackson lmmunoResearch Labs 711-605-152 AB_2492288
Alexa 647anti-guinnea-pig donkey Jackson lmmunoResearch Labs 706-606-148 AB_2340477
Biotinylated anti-chicken goat Vector Laboratories BA-9010 AB_2336114

Validation

All primary antibodies used in this study are commercially available,
the specificity and application were validated by the manufacturer, and omission of either primary or secondary antibodies resulted in a complete loss of signal.

Animals and other research organisms

Policy information about studies involving animals; ARRIVE guidelines recommended for reporting animal research, and Sex and Gender in Research

Laboratory animals

Experiments were carried out on 2-19 weeks old mice.

Wild animals

C57BL/6J (RRID:IMSR_JAX:000664),
P2Y12R-/- (B6;129-P2ry12tm1Dgen/H P2Y12R KO, Taconic #TF1881),
CX3CR1GFP/+, CX3CR1GFP/GFP (RRID:IMSR_JAX:005582)
Vglutl/cre/Gt(Rosa26)Sor[cre/cre] x
LSL_GRABATP_P2A_jRGECOla (TgTm) - C57B1/6J [flox/flox] (Biocytogen, China)
Mice were kept in a 12h dark/light cycle environment,
under controlled temperature and humidity with food and water ad libitum.

Reporting on sex

Mice of both sexes were used in the study.

Field-collected samples	*For laboratory work with field-collected samples, describe all relevant parameters such as housing, maintenance, temperature, photoperiod and end-of-experiment protocol OR state that the study did not involve samples collected from the field.*
Ethics oversight	All experimental procedures were in accordance with the guidelines set by the European Communities Council Directive (86/609 EEC) and the Hungarian Act of Animal Care and Experimentation (1998; XXVIII, Sect. 243/1998), approved by the Animal Care and Experimentation Committee of the Institute of Experimental Medicine and the Goverment Office of Pest County Department of Food Chain Safety, Veterinary Office, Plant Protection and Soil Conservation Budapest, Hungary under the numbers PE/EA/1021- 7/2019, PE/EA/673-7/2019 and Department of Food Chain Safety and Animal Health Directorate of Csongrad County, Hungary. Experiments were performed according to the EU Directive 2010/63/EU on the protection of animals used for scientific purposes, and reported in compliance with the ARRIVE guidelines.

Note that full information on the approval of the study protocol must also be provided in the manuscript.

Plants

Seed stocks	*Report on the source of all seed stocks or other plant material used. If applicable, state the seed stock centre and catalogue number. If plant specimens were collected from the field, describe the collection location, date and sampling procedures.*
Novel plant genotypes	*Describe the methods by which all novel plant genotypes were produced. This includes those generated by transgenic approaches, gene editing, chemical/radiation-based mutagenesis and hybridization. For transgenic lines, describe the transformation method, the number of independent lines analyzed and the generation upon which experiments were performed. For gene-edited lines, describe the editor used, the endogenous sequence targeted for editing, the targeting guide RNA sequence (if applicable) and how the editor was applied.*
Authentication	*Describe any authentication procedures for each seed stock used or novel genotype generated. Describe any experiments used to assess the effect of a mutation and, where applicable, how potential secondary effects (e.g. second site T-DNA insertions, mosiacism, off-target gene editing) were examined.*
