# Supplementary material for: Microglia contribute to neuronal synchrony despite endogenous ATP-related phenotypic transformation in acute mouse brain slices
Source: Nat Commun. 2024 Jun 26;15:5402. doi: 10.1038/s41467-024-49773-1 (PMC11208608; doi:10.1038/s41467-024-49773-1)
Supplement: Supplementary file 4 — Description of Additional Supplementary Files [file 41467_2024_49773_MOESM4_ESM.pdf]

Description of Additional Supplementary Files

Supplementary Movie Legends

Supplementary Movie 1. Time lapse XY (upper) and YZ (lower) views (maximum intensity projections) of a CX3CR1+/GFP microglia moving towards the surface (dotted line) in an acute brain slice. The cell body positions are indicated with colored dots. Note the extensive process outgrowth and cell body translocation. Scale bar: 10 µm.

Supplementary Movie 2. Time lapse volume view (from YZ view) of a CX3CR1+/GFP microglia with almost stationery cell body, showing still strong process redistribution in an acute brain slice. Scale bar: cell body diameter ~12 µm.

Supplementary Movie 3. Morphological changes of individual microglia cells within acute brain slices depicted by maximum intensity projections of XY views. The video starts 1h after slice preparation. Scale bar: 10 µm.

Supplementary Movie 4. Morphological differences of individual microglial cells at different timepoints during incubation within acute brain slices depicted by 3D reconstructions via morphological analysis tool (Heindl et al., 2018).

Supplementary Movie 5. ATP dynamics throughout the 5 hours period after slice preparation recorded from layers of the cortex and the hippocampus. Scale bar: 20 µm.

Supplementary Movie 6. Secondary mechanical injuries result in immediate, intense ATP release near the injury (bottom) compared to non-injured slices recorded ~4 hours after slice preparation in slices from both P74 or younger, P20 mice. Scale bar: 500 µm.

Supplementary Movie 7. ATP dynamics direct microglial process movement in a P2Y12Rand CX3CR1-dependent manner. Scale bar: 10 µm.
